# Development of a Prediction Model Based on RBF Neural Network for Sheet Metal Fixture Locating Layout Design and Optimization

**DOI:** 10.1155/2016/7620438

**Published:** 2016-04-04

**Authors:** Zhongqi Wang, Bo Yang, Yonggang Kang, Yuan Yang

**Affiliations:** The Ministry of Education Key Laboratory of Contemporary Design and Integrated Manufacturing Technology, Northwestern Polytechnical University, No. 127, Youyi Road (West), Xi'an 710072, China

## Abstract

Fixture plays an important part in constraining excessive sheet metal part deformation at machining, assembly, and measuring stages during the whole manufacturing process. However, it is still a difficult and nontrivial task to design and optimize sheet metal fixture locating layout at present because there is always no direct and explicit expression describing sheet metal fixture locating layout and responding deformation. To that end, an RBF neural network prediction model is proposed in this paper to assist design and optimization of sheet metal fixture locating layout. The RBF neural network model is constructed by training data set selected by uniform sampling and finite element simulation analysis. Finally, a case study is conducted to verify the proposed method.

## 1. Introduction

Sheet metal part is widely used in aviation industry and automotive industry due to its high strength and light weight [1]. However, sheet metal part always tends to deform at machining, assembly, and measuring stages during the whole manufacturing process because of its properties of thin wall, large size, and low rigidity. In order to constrain excessive sheet metal part deformation, Cai et al. [2] put forward an “*N*-2-1” (*N* > 3) locating principle, which indicates that the “*N*-2-1” locating principle is more suitable for sheet metal location than “3-2-1” principle. Apparently, in order to apply “*N*-2-1” locating principle to fixture locating layout design, the key is to find the optimal number of locators as well as their positions so as to reduce sheet metal part deformation.

To solve the problem above, many scholars and technicians carried out a lot of research. Kashyap and Evries [3] analyzed the clamping deformation of sheet metal part along the normal direction at the locating point by finite element method (FEM). After calculating the clamping deformation by FEM, Kaya and Chen [4, 5] established an optimization model to minimize the sheet metal part deformation and obtained the optimal locators position by genetic algorithm (GA). Similarly, after knowing the deformation by FEM, Liu et al. [6] determined the initial number and positions of the locators by adding locators on the datum plane at the position with the maximum deformation repeatedly until the deformation was reduced within the range of machining accuracy. Then, the final positions were optimized along the feed direction to reduce the maximum deformation of the workpiece during the entire milling process. Prabhaharan et al. [7] presented a fixture layout optimization method that used GA and ant colony algorithm separately to decrease the dimensional and form errors by FEM.

All the above papers calculated the concerned workpiece deformation by means of FEM for deformation control and, as a result, the evolutionary algorithm-based fixture layout optimization procedure has to involve thousands of computationally expensive finite element analysis. Therefore, in order to save computational time and improve the optimization efficiency, Hamedi [8] trained back propagation neural network by only a few finite element analysis (FEA) results to recognize the pattern between the clamping forces and state of contact in the workpiece-fixture system and the workpiece maximum elastic deformation. Vasundara et al. [9] applied back propagation neural network to approximate the relationship between the position of the fixturing elements and the workpiece elastic deformation and compared the performance of ANN and RSM. Selvakumar et al. [10] used back propagation neural network to describe the function relationship of the position of the locators and clamps and the maximum workpiece deformation and combined ANN with DOE to optimize the machining fixture layout. Selvakumar et al. [11] integrated GA with ANN to accomplish the optimal machining fixture layout. Lu and Zhao [12] built a back propagation neural network model to predict the deformation of the sheet metal workpiece under different fixture layouts and different fixture locator errors and applied genetic algorithm to the established ANN model to find the optimal position of the fourth fixture locator based on the “*N*-2-1” locating principle. Rex and Ravindran [13] developed a back propagation neural network to predict the elastic deformation of the workpiece-fixture system and proposed an integrated approach for the optimal fixture layout design. Qin et al. [14] constructed a back propagation neural network model depicting the mapping relationship between the multiple fixturing parameters and the clamping deformation of workpiece and developed a unified approach to multifixturing layout planning for thin-walled workpiece.

This paper, considering the low efficiency of the fixture locating layout optimization method by FEM, proposes an RBF neural network prediction model to assist design and optimization of sheet metal fixture locating layout. First, the method generates sample points by uniform sampling method and constructs the sample data set with the help of finite element analysis. Second, the nonlinear relationship between the sheet metal fixture locating layout and responding deformation is described by RBF neural network; that is, the prediction model is established. Finally, a case study is presented to demonstrate the proposed method, and the result shows that the method has preferable performance and higher prediction accuracy.

## 2. Problem Description

### 2.1. “*N*-2-1” Locating Principle for Sheet Metal Part

**Figure 1:**
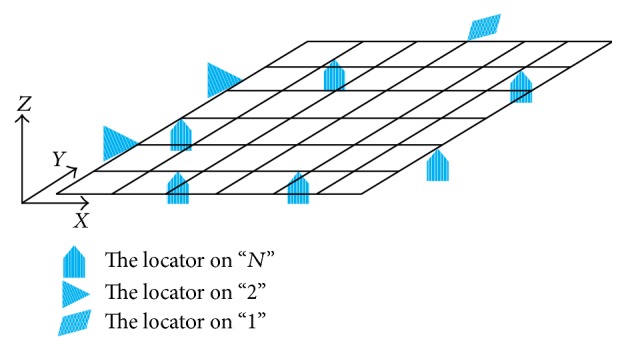
“*N*-2-1” locating principle of sheet metal part.

So as to prevent excessive deformation and supply more reinforcements for buckling prevention at machining, assembly, and measuring stages during the whole manufacturing process, sheet metal part is always under an overconstraint condition, which is the so-called “*N*-2-1” locating principle. The principle considers that there are “*N*” (*N* > 3) locating points on the primary datum plane of sheet metal part and “2” and “1” on the second and third datum plane, respectively.  Figure 1 shows a typical “*N*-2-1” principle, where 6 locators are required in order to support sheet metal on the primary datum plane to avoid excessive deflection. Meanwhile, the locator number “*N*,” which is always more than three, is determined by the dimensional specifications of sheet metal part. Obviously, the key problem of locating layout designing based on “*N*-2-1” principle is how to determine the number and position of “*N*,” that is, the fixture locating layout.

### 2.2. Fixture Locating Layout Optimization Model

By using “*N*-2-1” locating principle, the deformation of the main datum plane of sheet metal part along the normal direction can be reduced. In order to evaluate the quality of different fixture locating layout schemes, the normal deformation of all finite element nodes of the part is chosen as the evaluation function:

(1) $$\begin{array}{c} F\left(\;X\;\right)=\frac{\sum_{i=1}^{M}{w_{i}}^{2}\left(X\right)}{M}, \end{array}$$

where *F*(**X**) is the evaluation function for sheet metal deformation; **X** is the vector composed of fixture locating layout parameters; *M* is the number of the finite element notes in the sheet metal part; *w*_*i*_ is the normal deformation of the *i*th node.

In this paper, the finite element model of sheet metal fixture locating layout is established, so that the sheet metal deformation can be analyzed to train the neural network. And then, with the nonlinear mapping property of neural network, the prediction model of sheet metal deformation, meeting the need of general engineering, is suggested according to the limited training samples. Let *Ω*_*A*_ be the nodes set of finite element model of sheet metal part, and let fixture locating layout vector **X** be the design variable. **X** should satisfy the following constraints: (1)  **X** must be within the determined nodes set of finite element model of sheet metal part; (2) in each fixture locating layout scheme, any two locating points cannot coincide. Thus, the optimization model for sheet metal fixture locating layout scheme can be defined as

(2) Find X=x1,x2,…,xj,…,xN,Min FX=∑i=1Mwi2XM,s.t. xj∈ΩA, xi≠xj,

where **x**_*j*_ is the position vector of the *j*th locating point; *N* is the number of locating points.

## 3. Prediction Model for Sheet Metal Fixture Locating Layout

From the analysis above, it can be seen that when fixture locating layout parameters are given, we can use FEM to calculate the sheet metal deformation. However, as the fixture layout parameter varies, we cannot analyze the deformation one by one, considering that it is a demanding job and troublesome. To solve the problem, a prediction model for sheet metal deformation is built in the paper based on RBF neural network. What is more, by comparing with the BP neural network prediction model, the feasibility and superiority of RBF neural network prediction model are proved sufficiently [15].

### 3.1. BP (Back Propagation) Neural Network

**Figure 2:**
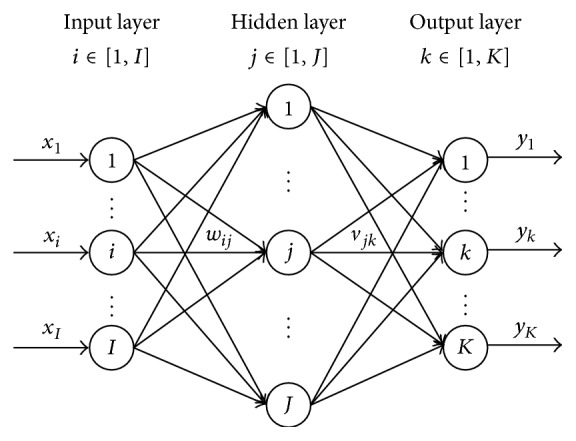
Network structure of BP neural network.

BP neural network is a feed-forward neural network with three or more layers, including input layer, hidden layer, and output layer. It has *I* input nodes, *J* hidden nodes, and *K* output nodes. Since it is proved that any multivariable function can be approximated to any desired degree of accuracy with a three-layer BP neural network, a three-layer BP neural network can be used in this paper to predict the sheet metal deformation given a certain fixture locating layout.  Figure 2 shows the network structure of BP neural network.

### 3.2. RBF (Radial Basis Function) Neural Network

**Figure 3:**
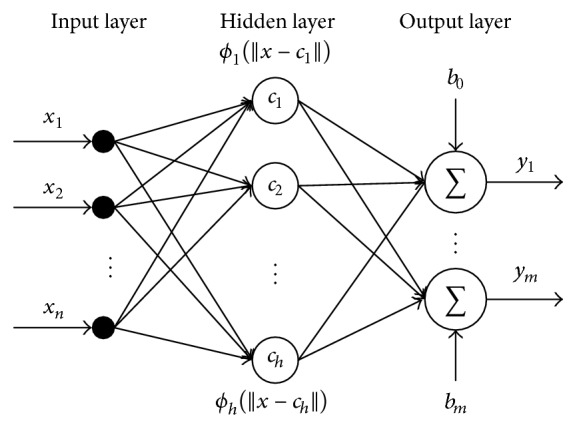
Network structure of RBF neural network.

RBF neural network is also a feed-forward neural network. It has *n* input layer nodes, *h* hidden layer nodes, and *m* output layer nodes. In RBF network, **x** = (*x* ^1^, *x* ^2^,…,*x* ^*n*^)^*T*^ ∈ *R* ^*n*^ is the input vector, and *ϕ*_*i*_(*∗*) is the activation function of hidden nodes, which is a Gaussian function in this paper. The hidden nodes in RBF network have local characteristics for input usually; that is, the farther away the input is from the center of the hidden node, the weaker effect the hidden node has on the input. Therefore, each hidden node in the RBF network has a data center *c*_*i*_, which determines that, for a specific input, there will be a specific number of neurons to be activated. *b*_0_,…, *b*_*m*_ are the offsets of output nodes. *y* = (*y*_1_,…,*y*_*m*_)^*T*^ is the network output.  Figure 3 shows the network structure of RBF neural network.

### 3.3. The Experimental Design for Training Data Set

In this section, the training sample points are selected by uniform sampling method and the responding deformation is calculated by FEM. Meanwhile, normalization of input data is needed so that those relatively large inputs are still within the region where the transfer function has a large gradient, which can improve identification precision of the neural network. The following formula can be used for normalization so that each sample data falls in [0,1]:

(3) $$\begin{array}{c} x_{i}^{\prime }=\frac{x_{i}-x_{min}}{x_{max}-x_{min}}, \end{array}$$

where *x*_*i*_′ is the *i*th input sample and *x*_min_ and *x*_max_ are the lower and upper sides of input sample, respectively.

## 4. The Flowchart of Building the Prediction Model

**Figure 4:**
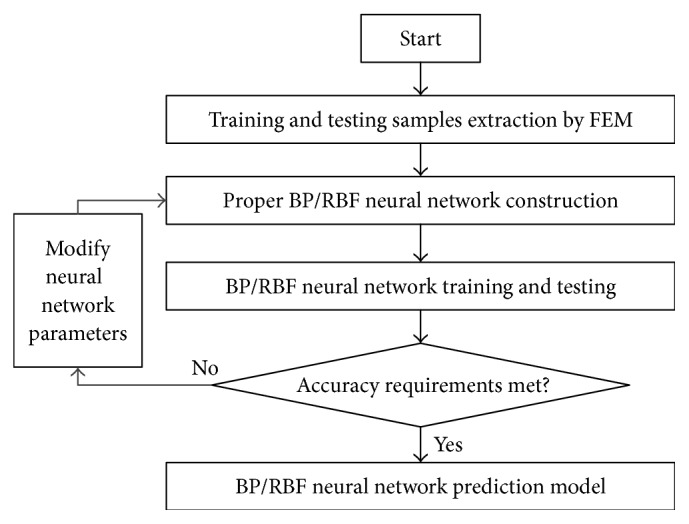
The flowchart of the prediction model for sheet metal fixture locating layout.

After the sample data is selected and normalized, the training and testing work for neural network can be conducted. Due to the nonlinear mapping relationship between the input and output, the initial weights play a great role in deciding whether the training work can achieve a local minimum or can converge. Therefore, evenly distributed decimal empirical value should be chose as the initial weights. Then, the above network is simulated and calculated with MATLAB, and the nonlinear mapping between the input and output is realized. The flowchart of the prediction model for sheet metal fixture locating layout is depicted in Figure 4.

## 5. Case Study

**Figure 5:**
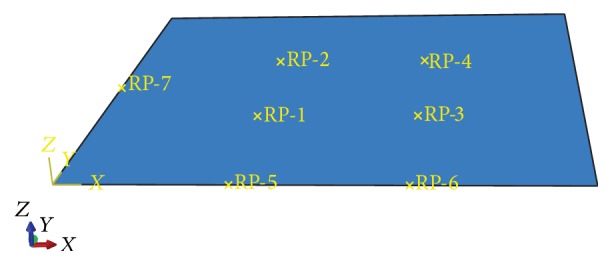
The initial fixture locating layout of the aluminum alloy sheet metal part.

In this section, the prediction model based on BP/RBF neural network for sheet metal fixture locating layout design and optimization is illustrated by an aluminum alloy sheet metal part, and its fixture locating scheme given “*N* = 4” is analyzed. As shown in Figure 5, the sheet metal has dimensions of 400 × 400 × 1 mm^3^, and the physical properties of material are listed in Table 1. The “*N* = 4” locating points on the primary datum plane are RP-1, RP-2, RP-3, and RP-4. The “2” locating points on the second datum plane are RP-5 and RP-6. And the “1” locating point on the third datum plane is RP-7. Set the coordinates of the fixed locating points RP-1, RP-2, RP-3, RP-5, RP-6, and RP-7 as (100, 100), (100, 300), (300, 100), (133, 0), (267, 0), and (0, 200). The locating point to be optimized is RP-4 and its coordinate is (*x*, *y*).

The training and testing data sets, as shown in Tables 2 and 3, are generated by uniform sampling method. And the normal deformation of each finite element note of the sheet metal under its deadweight is calculated by the commercial finite element software ABAQUS [16].

In summary, referring to the flowchart of the prediction model for sheet metal fixture locating layout, the prediction models based on BP neural network and RBF neural network are established separately with the help of MATLAB neural network toolbox [17]. In BP neural network structure, the input layer has two neurons (*I* = 2), which, respectively, represent the *x* and *y* coordinates of RP-4. The output layer has one neuron (*K* = 1), that is, the evaluation function value *F*(**X**) for sheet metal deformation. According to the general empirical formula *J* = 2 · *I* + 1 = 5, the hidden layer has five neurons (*J* = 5).

**Figure 6:**
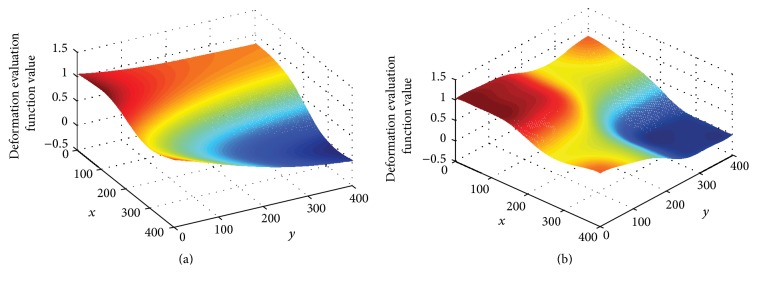
The response surfaces of BP and RBF neural network prediction models.

**Figure 7:**
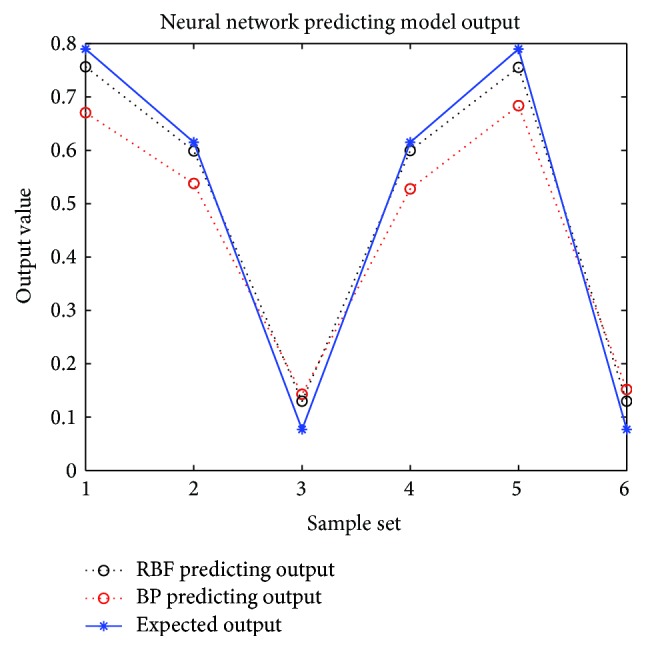
The output comparison between BP and RBF neural network prediction models.

Therefore, the response surface model describing the mapping relation between the fixture locating layout scheme and sheet metal part deformation can be established by fixture locating layout and the responding deformation evaluation function. In other words, given a locating layout scheme, the sheet metal part deformation can be obtained. The response surfaces of the neural network prediction models are shown in Figure 6. Finally, the output curves and the corresponding relative errors are shown in Figure 7 and Table 4.

## 6. Conclusions

In order to assist the design and optimization of sheet metal fixture locating layout, this paper establishes an RBF neural network prediction model to describe the mapping relationship between sheet metal fixture locating layout and responding deformation. The major contributions of this paper include the following:

1. A prediction model based on RBF neural network for sheet metal fixture locating layout design and optimization is developed, and the prediction accuracy meets the need of general engineering.
2. Once the proposed prediction model is applied to fixture locating layout optimization in the near future, it can replace the finite element simulation for sheet metal deformation. Thus, the calculation amount is reduced and therefore the efficiency of fixture locating layout design and optimization is improved.
3. Compared with the BP neural network trained and tested with the same sample sets, the RBF neural network based prediction model is of higher precision and more stable.

## Figures and Tables

**Table 1:** The physical properties of material.

Material properties	Value
Mass density	2.8 × 10^3^ kg/m^3^
Young's modulus	7.12 × 10^4^ MPa
Poisson ratio	0.33

**Table 2:** Training data set.

Number	1	2	3	4	5	6	7	8
Coordination	(0, 0)	(133, 0)	(267, 0)	(400, 0)	(0, 133)	(133, 133)	(267, 133)	(400, 133)
∑	1.0529	1.0549	0.6538	0.8257	0.9948	0.9839	0.5418	0.6421

Number	9	10	11	12	13	14	15	16

Coordination	(0, 267)	(133, 267)	(267, 267)	(400, 267)	(0, 400)	(133, 400)	(267, 400)	(400, 400)
∑	0.6518	0.5418	0.0201	0.0427	0.8273	0.6421	0.0427	0.0234

**Table 3:** Testing data set.

Number	1	2	3	4	5	6
Coordination	(40, 360)	(120, 280)	(240, 400)	(280, 120)	(360, 40)	(400, 240)
∑	0.7898	0.6150	0.0771	0.6150	0.7898	0.0771

**Table 4:** The relative errors of the prediction models.

Prediction models	Relative error
BP neural network prediction model	11.66%
RBF neural network prediction model	6.91%
